# Association Between Pharyngeal Airway Volume and Craniofacial Morphology in Skeletal Class I and Class II Adult Patients Assessed Using Cone Beam Computed Tomography

**DOI:** 10.7759/cureus.86634

**Published:** 2025-06-23

**Authors:** Wangonsana Rajkumari, Nungshinaro Tzudir, Altaf H Thekiya, Butool Zohra, Harsh Garg, Adesh Watane, Santosh Kumar, Seema Gupta

**Affiliations:** 1 Department of Orthodontics, Kothiwal Dental College and Research Centre, Moradabad, IND; 2 Department of Dentistry, Jain Clinic, Dimapur, IND; 3 Department of Orthodontics, Diamond Dental Care, Nanded, IND; 4 Department of Orthodontics, Al Badar Rural Dental College and Hospital, Kalaburagi, IND; 5 Department of Orthodontics, Triveni Institute of Dental Sciences, Hospital and Research Centre, Bilaspur, IND

**Keywords:** airway, malocclusion, pharyngeal, skeletal, volume

## Abstract

Introduction

This study aimed to determine whether pharyngeal airway volumetric differences exist between orthodontic subjects with skeletal Class I and Class II adult participants, and to assess the correlation between age, sex, and body mass index (BMI). The identification of these relationships might enhance orthodontic diagnosis, airway risk assessment, and treatment planning.

Materials and methods

A prospective observational study was conducted on 32 participants (aged 18-28  years) who were equally divided into dentoskeletal Class I (n = 16) and skeletal Class II with dental Class II Division 1 (n = 16) groups, each with a balanced sex distribution. Cone beam computed tomography (CBCT) scans were acquired using the Carestream 9300 Select (Carestream Dental LLC, Atlanta, GA) system with a field of view (FOV) of 10 × 10 cm, 90 kVp, 4  mA, and a voxel size of 180 µm. Pharyngeal airway volumes, such as nasopharyngeal (NPV), oropharyngeal (OPV), and total airway volume (TAV), were delineated using Carestream 3D imaging software (Carestream Dental, Rochester, NY). BMI was calculated from calibrated weight and height measurements. Intra- and inter-examiner reliability was confirmed (intraclass correlation coefficient (ICC) 0.89-0.94). Statistical analysis included the Shapiro-Wilk test, independent t-tests comparing anatomical volumes across malocclusion and sex subgroups, and Pearson correlations (α = 0.05).

Results

The mean age did not differ significantly between sexes or malocclusion types (p > 0.05). BMI was significantly higher in Class  I versus Class  II (25.02 ± 4.17 vs. 21.41 ± 3.62 kg/m²; p = 0.014), with no sex-based BMI differences (p = 0.474). An independent t-test analysis of airway parameters by sex revealed no statistically significant differences between males and females (p > 0.05). No significant correlations were identified between BMI and airway volume (p > 0.05) in either group. Class II patients exhibited statistically significantly lower NPV and TAV (p < 0.05). No significant differences were noticed in OPV between malocclusion groups (p > 0.05).

Conclusion

This study demonstrated that patients with Class II Division 1 malocclusion exhibited significantly reduced NPV and TAV compared to those with Class I malocclusion, highlighting the influence of craniofacial morphology on airway dimensions.

## Introduction

The pharyngeal airway, a tube-shaped structure extending from the nasal and oral cavities to the larynx, plays a critical role in respiration and influences craniofacial development. Spanning approximately 12-14 cm, it comprises three segments: nasopharynx, oropharynx, and laryngopharynx [1]. The nasopharynx, located posterior to the nasal cavity and superior to the soft palate, contains lymphatic tissues, such as adenoids, which can contribute to airway constriction when hypertrophied due to inflammation or infection, particularly in growing children. The oropharynx lies between the soft palate and the hyoid bone, while the laryngopharynx extends to the sixth cervical vertebra. Alterations in airway morphology have been linked to breathing patterns and craniofacial growth, prompting extensive research into their interrelationships [2].

The "soft tissue stretching hypothesis" proposed by Solow and Kreiborg [3] suggests that soft tissue pressure rather than dentition alone influences dental arch form, incisor positioning, and maxillomandibular growth patterns. For instance, head extension or mandibular rotation may stretch the surrounding muscles, leading to shorter dental arches and upright incisors, which are often observed in open-bite or long-face syndromes [4]. Similarly, Moss's functional matrix hypothesis posits that soft tissues guide hard tissue development, as evidenced by studies showing normalized craniofacial morphology in children post-adenoidectomy [5]. Experimental studies have demonstrated that nasal obstruction in humans induces mandibular lowering and muscle relaxation, potentially contributing to excessive vertical alveolar growth and long-face syndrome due to reduced masticatory muscle activity [6,7].

Pharyngeal airway dynamics are also critical in conditions such as obstructive sleep apnea (OSA), which is characterized by recurrent airway collapse during sleep. Factors such as obesity and craniofacial deformities, including reduced airway volume, exacerbate OSA severity [8]. Sexual dimorphism in airway volume remains debated, with some studies indicating larger nasopharyngeal volumes in males [9], whereas others found no significant differences in total airway capacity [10].

Historically, airway assessments have relied on two-dimensional (2D) lateral cephalometric radiographs, which limit the evaluation of complex three-dimensional (3D) structures [11]. Angle’s early observations of restricted airways in Class II dentofacial deformities sparked interest in airway research; however, the limitations of 2D imaging have hindered comprehensive analysis. The advent of cone beam computed tomography (CBCT) has revolutionized airway evaluation by providing detailed 3D reconstructions, enabling precise volumetric, angular, and linear measurements of both soft and hard tissues. The ability of CBCT to isolate internal structures, such as the airway, has facilitated advanced studies on its morphology and relationship with malocclusion types [12].

This study aimed to compare pharyngeal airway volumes across different malocclusion groups, specifically Class I and Class II Division 1, using CBCT. By assessing nasopharyngeal (NPV), oropharyngeal (OPV), and total airway volumes (TAV) and exploring correlations with age, sex, and body mass index (BMI), this study sought to elucidate the interplay between airway morphology, craniofacial structure, and malocclusion, contributing to improved orthodontic diagnosis and treatment planning.

## Materials and methods

Study design and setting

This prospective observational study was conducted at the Department of Orthodontics and Dentofacial Orthopaedics, Kothiwal Dental College and Research Centre, Moradabad, from May 2023 to December 2024. The study was approved by the Institutional Ethics and Review Board (KDCRC/IERB/04/2023/28). CBCT scans obtained as part of the diagnostic records for assessing impacted teeth or orthodontic treatment needs were used. All the patients provided informed consent for the use of their data for scientific purposes. The study followed the principles of the Declaration of Helsinki.

Sample size calculation

The sample size was determined using G*Power software version 3.1.9.2 (Heinrich-Heine-Universität Düsseldorf, Düsseldorf, Germany). Based on an effect size of 0.94, a 5% significance level, a 95% confidence level, and an 80% power, a minimum sample size of 30 was calculated [1]. To ensure robustness, 32 patients were recruited for the study.

Sample selection

The study employed a single-blinded design, in which the patients were blinded to the group allocation. The patients were selected based on specific inclusion and exclusion criteria. Inclusion criteria were as follows (Table 1).

**Table 1:** Selection criteria for the study patients

S. no.	Selection	Criteria
1	Inclusion criteria	Age range of 18-28 years
		Presence of all teeth except permanent third molars
		Angle’s Class I malocclusion with Class I skeletal base (0^0^ ≤ ANB < 4^0^) or Class II Division 1 malocclusion with Class II skeletal base (ANB ≥ 4^0^). ANB angle is the angle between the deepest point in the concavity of maxillae (A point), mandible (B point), and the most prominent point on frontonasal suture (N point) [2]
		Vertical craniofacial growth of the mandible with respect to the cranial base was taken as growth pattern. The mesofacial patients were selected. For that, the Jarabak’s analysis facial height ratio was used (mesofacial = 61% ± 2%) [2].
2	Exclusion criteria	History of prior orthodontic treatment or orthognathic surgery
		Chronic mouth breathing
		Pharyngeal pathologies such as adenoid hypertrophy or tonsillitis
		Cleft lip and/or palate
		Adenoid or tonsil removal
		Medical conditions affecting weight, height, or body composition

Methodology

A total of 32 patients were categorized into dentoskeletal Class I (n = 16) and skeletal Class II with Angle's Class II Division 1 (n = 16) groups based on ANB angle and Angle’s malocclusion classification. Each group was further subdivided into male and female groups.

Patient preparation and CBCT procedure

CBCT scans were performed in the Department of Oral Medicine and Radiology using a Carestream 9300 Select Machine (Carestream Dental LLC, Atlanta, GA). The patients were instructed to remove all metallic objects from the head and neck region. Personal radiation barrier protection was provided, and head stabilization was achieved using a restraining strap and laser pointer to align the head of the patient in such a way that the Frankfort horizontal (FH) plane was parallel to the floor. The patients were instructed to remain still, avoid swallowing, and breathe normally during the scan. The exposure parameters were as follows: field of view (FOV), 10 × 10 cm; peak kilovoltage (kVp) 90, milliampere (mA) 4.0, voxel size, 180 µm; and exposure time, 20 seconds.

Image reconstruction

**Figure 1:**
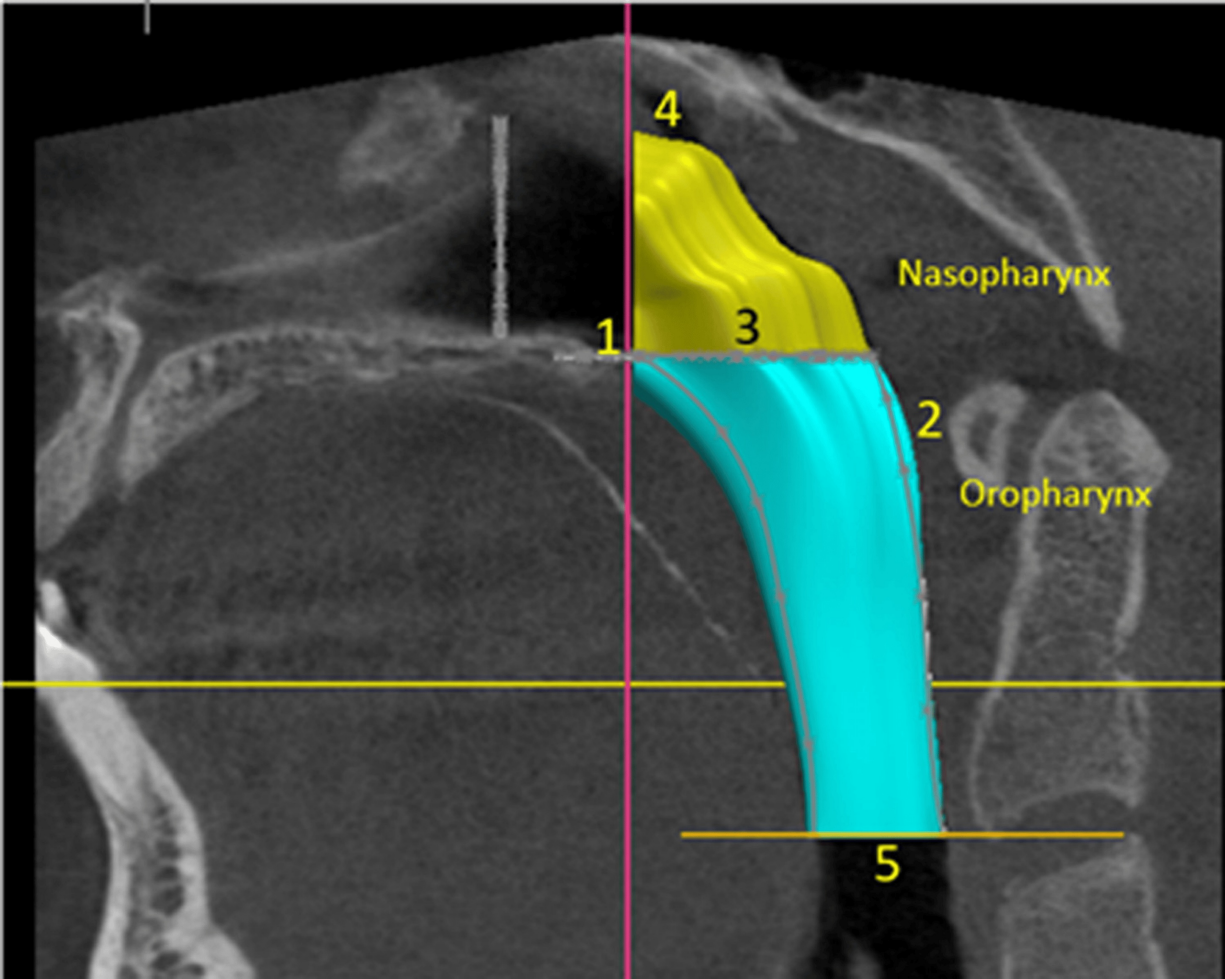
Three-dimensional (3D) reconstruction of airway Airway boundaries were defined as follows: (1) anterior border: a vertical plane through the posterior nasal spine, perpendicular to the palatal plane; (2) posterior border: the posterior pharyngeal wall; (3) inferior nasopharyngeal border: the palatal plane; (4) superior nasopharyngeal border: a line from the posterior nasal spine to the upper limit of the oropharynx; and (5) inferior oropharyngeal border. This figure represents a volumetric assessment of the pharyngeal airway in a patient from the study and is published with the patient's consent.

CBCT projection data were processed using the Feldkamp-Davis-Kress algorithm to generate a volumetric dataset. Images were formatted into Digital Imaging and Communications in Medicine (DICOM) files and reconstructed into a continuous slice thickness of 0.76 µm. All measurements were performed by two calibrated examiners (Altaf Hussain Thekiya, Butool Zohra) to minimize human errors (Figure 1).

Airway assessment

Airway volume measurements, including NPV, OPV, and TAV, were conducted using the Carestream 3D imaging software (Carestream Dental, Rochester, NY). Images were adjusted to the tissue threshold to isolate soft tissues, followed by hard tissue visualization and orientation in three planes to minimize errors from non-standardized head positions. The palatal plane (anterior nasal spine as ANS to posterior nasal spine as PNS) was used as a reference, aligned parallel to the global horizontal plane in the sagittal view, and perpendicular to the axial view. Airway boundaries were defined as follows: (1) anterior border: a vertical plane through the posterior nasal spine, perpendicular to the palatal plane; (2) posterior border: the posterior pharyngeal wall; (3) inferior nasopharyngeal border: the palatal plane; (4) superior nasopharyngeal border: a line from the posterior nasal spine to the upper limit of the oropharynx; and (5) inferior oropharyngeal border: a line parallel to the palatal plane through the most anterior inferior point on the third cervical vertebra [1]. The NPV was calculated as the volume between the superior nasopharyngeal border and the palatal plane. The OPV was calculated as the volume between the palatal plane and the inferior oropharyngeal border. The TAV was determined as the sum of the NPV and OPV.

Pharyngeal airway volume calculation

The volume calculation process involved the following steps: (1) Import CBCT data into Radiant software (Medixant, Warsaw, Poland); (2) extract images and stereolithography (STL) files from the CBCT data; (3) import the extracted images and STL files into SOLIDWORKS (Dassault Systèmes, Vélizy-Villacoublay, France); (4) trace the images and STL files within SOLIDWORKS; (5) create sketches and features based on the traced data; (6) generate a regular volume using the Loft feature in SOLIDWORKS; and (7) measure the volumes of the generated model.

Anthropometric measurements

BMI was calculated from weight and height measurements. Weight was measured to the nearest 0.1 kg using a calibrated digital scale, with participants wearing light clothing and no shoes. Height was measured to the nearest 0.1 cm using a stadiometer, with participants standing upright, aligning their head in the Frankfort horizontal plane.

Validity and reliability testing

Intra- and inter-examiner reliability was assessed by repeating measurements on 12 samples after a two-week interval, yielding an intraclass correlation coefficient (ICC) of 0.89-0.94, indicating good to excellent reliability.

Statistical analysis

Statistical analysis was done by Stata Statistical Software Release 18 (StataCorp LLC, College Station, TX). All the statistical analysis was performed by a statistician (Adesh Watane) who was provided with coded data. Descriptive statistics (mean and standard deviation) were calculated for all variables and are presented in tables and graphs. The Shapiro-Wilk test confirmed normal data distribution, allowing the use of parametric tests. Independent t-tests compared NPV, OPV, and TAV between the Class I and Class II Division 1 groups. Statistical significance was set at p ≤ 0.05. Pearson’s correlation coefficient was used to assess correlations among age, sex, BMI, and pharyngeal airway volume.

## Results

The study included an equal population of males (16, 50%) and females (16, 50%) in both malocclusion groups. The independent t-test analysis of demographic characteristics revealed no significant differences in age between males (20.56 ± 2.90 years) and females (20.19 ± 1.91 years) (p = 0.668) or between Class I (20.44 ± 2.48 years) and Class II malocclusion groups (20.31 ± 2.44 years) (p = 0.887). However, BMI differed significantly (p = 0.014) between malocclusion classes, with Class I patients (25.02 ± 4.17 kg/m²) having higher BMI than Class II (21.41 ± 3.62 kg/m²). Sex-based BMI differences (males: 23.76 ± 4.51 kg/m²; females: 22.66 ± 4.06 kg/m²) were non-significant (p = 0.474). These findings suggest that the malocclusion class may be associated with BMI, whereas age and sex showed no notable influence in this population (Table 2).

**Table 2:** Analysis of demographic characteristics of study population with independent t-test n (%) is the frequency of samples in each group. Data are presented in the form of mean and standard deviation (SD). *p-value < 0.05: significant level

Variables	Category	n (%)	Age (years)				BMI (kg/m^2^)			
			Mean ± SD	Mean difference	t-value	p-value	Mean ± SD	Mean difference	t-value	p-value
Sex	Male	16 (50)	20.56 ± 2.90	0.38	0.43	0.668	23.76 ± 4.51	1.10	0.73	0.474
	Female	16 (50)	20.19 ± 1.91				22.66 ± 4.06			
Malocclusion	Class I	16 (50)	20.44 ± 2.48	0.13	0.14	0.887	25.02 ± 4.17	1.38	2.62	0.014*
	Class II Division 1	16 (50)	20.31 ± 2.44				21.41 ± 3.62			

An independent t-test comparing the airway parameters between the malocclusion groups revealed significant differences. Class II Division 1 exhibited significantly lower TAV (p = 0.046) and NPV (p = 0.004) than Class I. However, the OPV was not significantly different (p = 0.169). These findings suggested that skeletal Class II malocclusion was associated with decreased nasal and total airway dimensions, possibly reflecting compensatory pharyngeal obstructions, while OPV remained unaffected (Table 3).

**Table 3:** Comparison of airway parameters between both malocclusion groups with an independent t-test. n (%) is the frequency of samples in each group. Data are presented in the form of mean and standard deviation (SD). *p-value < 0.05: significant level

Parameter	Groups	n (%)	Mean ± SD	Mean difference	t-value	p-value
Total airway volume (mm^3^)	Class I	16 (50)	20695.21 ± 5559.52	3152.19	2.09	0.046*
	Class II Division 1	16 (50)	17543.03 ± 2367.43			
Oropharyngeal volume (mm^3^)	Class I	16 (50)	15324.07 ± 4613.11	1769.14	1.41	0.169
	Class II Division 1	16 (50)	13554.94 ± 1985.73			
Nasopharyngeal volume (mm^3^)	Class I	16 (50)	5371.20 ± 1368.32	1383.11	3.11	0.004*
	Class II Division 1	16 (50)	3988.09 ± 1134.22			

An independent t-test analysis of airway parameters by sex revealed no statistically significant differences between males and females. TAV showed a mean difference of 1994.76 mm³ (p = 0.215). Similarly, OPV differed by 1435.06 mm³ (p = 0.267) and NPV by 559.64 mm³ (p = 0.273), with all p-values > 0.05. These findings suggested that sex did not significantly influence the airway parameters (Table 4).

**Table 4:** Comparison of airway parameters between sexes with an independent t-test n (%) is the frequency of samples in each group. Data are presented in the form of mean and standard deviation (SD). *p-value < 0.05: significant level

Parameter	Groups	n (%)	Mean ± SD	Mean difference	t-value	p-value
Total airway volume (mm^3^)	Female	16 (50)	4399.83 ± 1339.92	1994.76	1.27	0.215
	Male	16 (50)	4959.47 ± 1488.82			
Oropharyngeal volume (mm^3^)	Female	16 (50)	13721.97 ± 3081.92	1435.06	1.13	0.267
	Male	16 (50)	15157.04 ± 4036.91			
Nasopharyngeal volume (mm^3^)	Female	16 (50)	18121.74 ± 3988.50	559.64	1.12	0.273
	Male	16 (50)	20116.50 ± 4876.68			

Pearson correlation analysis revealed no statistically significant relationships between airway volumetric parameters and BMI in either malocclusion group (all p > 0.05). These findings suggested that airway dimensions and BMI varied independently in both malocclusion types, indicating that craniofacial morphology rather than body mass might be the primary determinant of airway volume characteristics in these populations (Table 5).

**Table 5:** Pearson correlation between volumetric parameters of Class I and Class II Division 1 groups Very weak correlation: 0.0<∣r∣<0.20. Weak: 0.2≤∣r∣<0.4. Moderate: 0.4≤∣r∣<0.6. Strong: 0.6≤∣r∣<0.8. Very strong: 0.8≤∣r∣≤1. *p-value < 0.05: significant level

Class II Division 1	Correlation	Class I			
		Body mass index	Nasopharyngeal volume	Oropharyngeal volume	Total airway volume
Body mass index	r-value	-0.27	0.21	-0.17	-0.09
	p-value	0.31	0.425	0.533	0.749
Nasopharyngeal volume	r-value	0.04	-0.26	0.01	-0.06
	p-value	0.871	0.322	0.972	0.834
Oropharyngeal volume	r-value	-0.13	0.17	0.06	0.09
	p-value	0.629	0.533	0.825	0.737
Total airway volume	r-value	-0.09	0.01	0.06	0.05
	p-value	0.745	0.958	0.84	0.856

## Discussion

The present study evaluated and compared pharyngeal airway volumes between the skeletal Class I and skeletal Class II with Angle's Class II Division 1 malocclusion groups using CBCT. These findings provide key insights into how craniofacial morphology influences airway dimensions and offer valuable implications for orthodontic diagnosis and treatment planning. The study revealed that patients with Class II Division 1 malocclusion exhibited a significantly smaller NPV and TAV than those with Class I malocclusion. Specifically, NPV was markedly reduced in the Class II group (p = 0.004), which is consistent with several prior studies that reported constricted nasopharyngeal spaces in individuals with retrognathic mandibles, a common trait of Class II skeletal patterns [1,2,13].

The reduced airway dimensions in Class II cases may be attributed to the posterior positioning of the mandible, which can result in the tongue and associated soft tissues encroaching into the airway space. This positional discrepancy potentially narrows the nasopharyngeal and oropharyngeal passages, leading to reduced overall pharyngeal airway volume [2,13]. Kim et al. [14] and Alves et al. [15] previously identified these anatomical restrictions in Class II individuals, corroborating the present findings.

Although the OPV was lower in Class II subjects, the difference was not statistically significant (p = 0.169). The absence of a significant difference could be attributed to compensatory mechanisms in the soft tissue, such as altered tongue posture and pharyngeal wall compliance, which might help maintain functional airway patency despite the skeletal discrepancies [3]. The wide range of OPV values, particularly within the Class I group, also indicates substantial inter-individual variability, possibly influenced by soft tissue thickness, posture, and muscle tone [3,4]. This finding is in contrast to that of Cabral et al. [16], who reported a small OPV in Class II patients.

The TAV was significantly lower in Class II patients (p = 0.046). This finding supports earlier studies that have established Class II skeletal morphology as a predisposing factor for smaller upper airway dimensions and potentially compromised respiratory function [1,2,13-15]. El et al. [17] found significantly smaller airway volumes in Class II individuals than in Class I and III groups, highlighting how mandibular retrusion adversely affects the airway space. Clinically, this finding emphasizes the importance of considering airway volume during treatment planning for Class II malocclusions. Mandibular advancement appliances or orthognathic surgery not only correct sagittal discrepancies but may also enhance airway volume and reduce the risk of OSA symptoms [18].

Although males in this study exhibited higher mean values for NPV, OPV, and TAV, these differences were not statistically significant. This finding is consistent with that of Ceylan and Oktay [19], who reported that sex did not substantially influence pharyngeal dimensions. Similarly, Handelman and Osborne [20] emphasized that variations in airway morphology are more likely to be due to skeletal growth and orthodontic interventions than to biological sex.

While some studies have identified sexual dimorphism in airway volumes, especially during growth phases [21,22], the absence of significant sex-based differences in the adult sample of our study may reflect stabilization of airway anatomy after adolescence. Thus, in a narrow age range (18-28 years), skeletal morphology appears to have a greater impact on airway volume than does sex.

Interestingly, the current study found that Class I participants had a significantly higher BMI than Class II participants (p = 0.014), although airway volumes were larger in Class I. However, no significant correlation was observed between BMI and airway volume in the malocclusion group. This suggests that while BMI differences exist between malocclusion types, BMI itself may not be a strong independent predictor of airway volume in this relatively healthy adult population [23].

In contrast, some studies, such as that by Genta et al. [24], have proposed a link between increased BMI and upper airway collapsibility owing to fat deposition around the pharyngeal walls. The lack of correlation in this study may be due to the exclusion of participants with obesity-related airway pathology or because the BMI range in this sample was not broad enough to elicit detectable anatomical changes.

No significant correlations were found between age (within the 18-28 years range) and any airway volume parameters. This was an expected finding, as airway growth typically stabilizes during late adolescence. Studies have shown that major changes in airway size occur during childhood and adolescence, with only minor modifications occurring in early adulthood [25,26]. Therefore, using a narrow and mature age group enhanced the internal validity by eliminating age as a confounding variable.

Clinical implications

The results of this study reinforce the notion that skeletal morphology significantly influences upper airway dimensions. Class II malocclusions, characterized by retruded mandibles, are more likely to be associated with reduced NPV, and TAV predisposing individuals to compromised airway function. Therefore, understanding these anatomical relationships is crucial to clinicians. Orthodontists and maxillofacial surgeons should consider airway health when planning treatment strategies involving mandibular retraction, orthognathic surgery, or extraction protocols. Tailoring treatment to preserve or enhance airway space, particularly in Class II patients, could help mitigate the risk of post-treatment airway obstruction or OSA. The use of CBCT and volumetric analysis software enables precise 3D visualization and measurement of airway volumes, offering a more comprehensive understanding than traditional cephalometric analyses. This finding strengthens the argument for incorporating airway evaluations into routine orthodontic assessments, especially in patients with skeletal discrepancies.

Study strengths and limitations

A notable strength of this study is the use of CBCT-based 3D volumetric analysis, which provides superior anatomical details compared to linear or 2D methods. Furthermore, controlling for variables such as age, growth pattern (average growers only), and prior treatment history enhances the internal validity.

However, this study had some limitations. The sample size was relatively small (n = 32), limiting the generalizability of our findings. Moreover, the cross-sectional design precluded causal inferences regarding changes in the airway volume over time or in response to treatment. The influence of dynamic factors, such as head posture, respiratory phase, and neuromuscular tone, which could affect airway measurements, was not considered. Patients with skeletal Class III and Class II Division 2 were not considered.

Future directions

Future studies should consider larger, more diverse samples and possibly longitudinal designs to assess airway changes over time or after orthodontic or surgical interventions. Integrating functional assessments, such as airflow dynamics or sleep studies, could provide a more comprehensive understanding of how volumetric changes affect clinical outcomes.

## Conclusions

This study demonstrated that patients with skeletal Class II with Angle's Class II Division 1 malocclusion exhibited significantly reduced NPV and TAV compared to those with dentoskeletal Class I malocclusion, highlighting the influence of craniofacial morphology on airway dimensions. Although differences in OPV were observed, they did not reach statistical significance, suggesting variability due to soft tissue and individual anatomical factors. No significant associations were found between airway volume and age, sex, or BMI within the studied age group (18-28 years), underscoring the predominance of skeletal patterns over other variables in determining airway space. These findings emphasize the need to incorporate airway assessment into orthodontic diagnosis and treatment planning, particularly in Class II cases, to prevent potential functional airway compromise.
